# Root Morphology and Canal Configuration of First and Second Maxillary Molars in a Selected Iranian Population: A Cone-Beam Computed Tomography Evaluation 

**DOI:** 10.22037/iej.v12i3.13708

**Published:** 2017

**Authors:** Abbasali Khademi, Asieh Zamani Naser, Zahra Bahreinian, Mojdeh Mehdizadeh, Mojtaba Najarian, Saber Khazaei

**Affiliations:** a *Dental Research Center, Department of Endodontics, Dental School, Isfahan University of Medical Sciences, Isfahan, Iran; *; b *Dental Implants Research Center, Radiology Research Committee, Department of Radiology, Dental School, Isfahan University of Medical Sciences, Isfahan, Iran; *; c *Department of Radiology, Dental School, North Khorasan University of Medical Sciences, Bojnourd, Iran*

**Keywords:** Cone-Beam Computed Tomography, Maxillary Molar, Mesiobuccal Canal, Root Canal Configuration

## Abstract

**Introduction:**

The aim of this investigation was to evaluate root canal morphology of maxillary first and second molars and also to assess the prevalence and morphology of the second mesiobuccal canal (MB2) in these teeth, using cone-beam computed tomography (CBCT).

**Methods and Materials:**

In this cross-sectional study, the total of 470 CBCT images from the archive of Radiology Department of Isfahan University of Medical Sciences (IUMS), Iran, was evaluated and 295 images were selected. The number of roots, and canal configuration were determined based on Vertucci’s classification system. The data was analyzed using SPSS 20, and *P*-values less than 0.05 were considered significant.

**Results:**

A total of 295 images from 295 patients (165 females and 130 males), including 389 maxillary first (197 right and 192 left) and 460 maxillary second (235 right and 225 left) molars were evaluated. The prevalence of MB2 canals were 70.2% and 43.4% in the maxillary first and second molars, respectively. The most common type of Vertucci’s classification was type II (53.1%), followed by type I.

**Conclusion:**

The second mesiobuccal canal was present in almost two thirds of first and less than half of second molars. The morphology and canal configuration of a maxillary molar can almost predict the morphology of contralateral molar. However, it does not relate to the ipsilateral molar.

## Introduction

Proper endodontic treatment is defined as cleaning, shaping and disinfection of the root canal system. Subsequently, a three-dimensional filling can be provided. Being aware of the root canal morphology, is an essential stage in achieving this goal. Failure in determining all the root canals can result in insufficient endodontic treatment [1, 2]. Navigation of the second mesiobuccal (MB2) canal in maxillary molars has always been an endodontic challenge for clinicians [3]. MB2 is the most commonly missed canal during endodontic treatment [4, 5]. It has been demonstrated that tooth morphology differs among different ethnic populations [6, 7]. Prevalence of MB2 canal of maxillary molars has been reported 52%, 63.59% and 42.63% in Chinese [8], Korean [6], and Brazilian [9] population, respectively. According to the result of a systematic review, the MB2 canal was present in 59.32% of the teeth. In 58.45% of these teeth it was a separate canal with distinct orifice and apical foramen (Vertucci’s type IV) [10].

Several methods have been used to declare the presence and morphology of MB2 canal [11]. Cone-beam computed tomography (CBCT) has been introduced as a reliable method for detection of the root canals morphology [7, 12, 13]. Some studies showed that it is the most accurate technique for this purpose [14-16].

The aim of the present study was to evaluate the number of roots, prevalence and morphology of MB2 in maxillary first and second molars using CBCT.

## Materials and Methods

In this cross-sectional study, a total of 470 CBCT images from the archive of Radiology Department, School of Dentistry, Isfahan University of Medical Sciences (IUMS), Iran, were evaluated. The Regional Bioethics Committee affiliated to IUMS approved the study protocol. The CBCT images had been taken from February 2010 to February 2015 for different diagnostic and treatment purposes.

The radiographs were obtained by Galileos (Sirona Dental Systems Inc., Bensheim, Germany), with 85 kVp, 42 mA, 150 µm voxel size, and 150×150 or 75×150 mm field of view. SIDEXIS XG software version 3.7 (Sirona Dental System GmbH, Germany) was used to evaluate the images. Images were displayed in a semi dark room with a 21.3-inch EIZO LCD (Nano Corporation, Ishikawa, Japan) with the resolution of 1600×1200 pixels.

Axial images were used as the basis of the analysis. The findings were then confirmed by evaluating the Sagittal and coronal images. Images of adult patients (more than 18 years of age) with at least one maxillary first or second molars present were assessed to find molars without root canal therapy, posts, crowns, under developed or abnormal roots, or root fractures. Out of 470 CBCT images, 295 images of 295 patients (165 females and 130 males) with the mean age of 42.1±1.6 years were enrolled in this study. 

**Figure 1:**
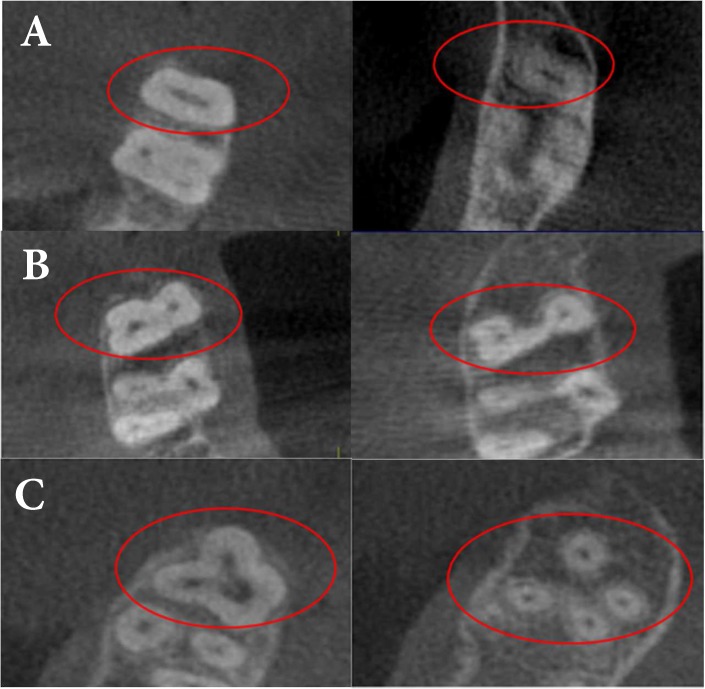
Assessing the number of the roots and canals in coronal sections of a maxillary molar: *A)* A single root; *B)* Two roots and; *C)* Four roots

**Figure 2:**
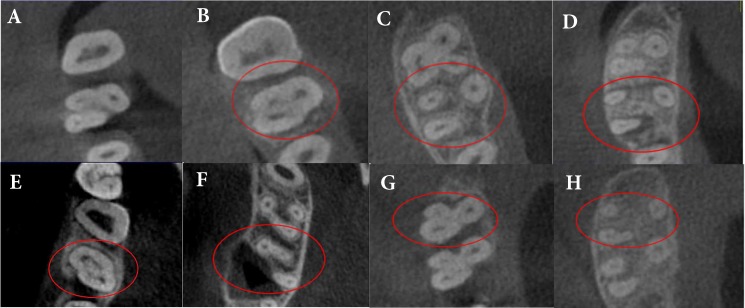
Canal morphology of mesiobuccal root in axial sections: *A)* Coronal and; *B)* Apical third in type I; *C)* Coronal and; *D)* Apical third in type II; *E)* Coronal and; *F)* Apical third in type IV; *G)* Coronal and; *H)* Apical third in type V Vertucci canal in mesiobuccal root

Two oral and maxillofacial radiologists were trained and calibrated for evaluating the classification criteria. Two observers evaluated the images together to come to a mutual agreement. Cases of disagreements between the two examiners were evaluated by a third radiologist to come to a certain diagnosis. The number of the roots, number of canals in each root, and the morphology of canals according to Vertucci’s classification system were evaluated. Moreover, the canal morphology was compared to the contralateral and ipsilateral molars (Figures 1 and 2). 

The data was analyzed using SPSS software version 20 (Statistical Package for Social Science software; SPSS Inc., Chicago, IL, USA). The *chi*-square test was used to compare the distribution of MB2 canal between genders, and Kendall Coefficient of Concordance was used to evaluate the prevalence of MB2 canal in contralateral molars as well as in ipsilateral first and second molars in each patient. *P*-value less than 0.05 was considered as significant level.

## Results

Out of 470 CBCT images, 295 images from 295 patients (including 165 female and 130 male patients) were included in this study that provided images of 389 maxillary first (197 on right and 192 on left side) and 460 maxillary second (235 right and 225 left) molars.

***Number of roots:*** In general, 93.5% of teeth had three roots. Table 1 demonstrates the prevalence of single-, two-, three- and four-rooted maxillary first and second molars. Kendall Coefficient of Concordance revealed a relatively strong positive association in the number of roots and canals between contralateral molars (Kendall’s coefficient of concordance=0.5). However, the same association was not seen between ipsilateral molars (Kendall’s coefficient of concordance=0.28).

***Prevalence of MB2: ***The prevalence of MB2 canal was 70.2% and 43.4% in the maxillary first and second molars, respectively. There was no significant difference between two genders in the prevalence of MB2 in maxillary first molars (*P*=0.825). However, in the maxillary second molars, more MB2 canals were found in male patients which was statistically significant (*P*=0.009). 

**Table 1:** Comparison of the N (%) of fracture resistance values between the two groups using independent t-test

	**Single-rooted**	**Double-rooted**	**Triple-rooted**	**Quad-rooted**	**Total**
**First molar**	0 (0)	1 (0.25)	388 (99.75)	0 (0)	389 (100)
**Second molar**	22 (4.7)	29 (6.3)	406 (88.2)	3 (0.6)	460 (100)

**Table 2:** The prevalence and N (%) of different morphologies in the mesiobuccal canal according to Vertucci classification

	**Definition**	**First molar **	**Second molar **
**Type I**	A single canal in a root	116 (29.8)	265 (57.6)
**Type II**	Two canals at the orifice merge to end into a single apical foramen	269 (69.1)	181 (39.3)
**Type IV**	Two separate canals in a root	3 (0.77)	8 (1.73)
**Type V**	A single canal at the orifice, branches and ends into two separate apical foramina	1 (0.25)	6 (1.3)
**Total**		389 (100)	460 (100)

***Classification of morphology in mesiobuccal root: ***In first molars the most common type of Vertucci’s classification was type II (53.1%), followed by type I (29.8%). In second molars, type I and II were seen in 57.6% and 39.3% of teeth, respectively. Only types I, II, IV and V of Vertucci’s classification were found in the present study (Table 2).

## Discussion

This study evaluated the number of roots, prevalence and morphology of MB2 canals in maxillary first and second molars in a selected Iranian population from Isfahan province, Iran from February 2010 to February 2015. In the present study high resolution CBCT images with the voxel size of 150 µm were used. Axial images were used as the basis of the analysis because axial view can show all the roots and canals in the same cut, therefore is suitable for counting the number of the roots, and also the number of canals in a root. Furthermore, the canal morphology can be evaluated in the axial sections moving from pulp chamber to apices [17]. 

Owing to clinical importance of determining MB2 canals through endodontic treatment, several studies have used *ex vivo* methods such as direct visualization, visualization under microscope, clearing, or sectioning for detecting MB2 canals [18-20]. CBCT is a relatively new method which has been shown to have excellent accuracy that provides a non-invasive procedure [21]. This method can be used in either *in vivo* or *ex vivo* studies. 

In the present study the prevalence of MB2 canal was 70.2% and 43.4% in the maxillary first and second molars, respectively. Rouhani* et al *[4] evaluated the root canal morphology of 125 first and 125 second maxillary molars of Iranian population and reported that three roots were found in 97% and 89% of first and second maxillary molars, respectively, and 53% and 20% first and second maxillary molars had MB2 canal, respectively. Most of teeth were three rooted as in the present study. However, the prevalence of MB2 canal was about 20% lower than the present study in either first or second molars. These differences can be as results of different ethnic subgroups, or differences in the design of the evaluation. 

A review study evaluated the use of CBCT for investigating the mesiobuccal root in first maxillary molar in 2013 [7]. They reported that in the total of 1964 first molars, the prevalence of MB2 canal was nearly 60%. In the present study the prevalence was 70%, which was higher than other studies. In addition, out of 1741 first molars, 38% had two separate canals in one root (type IV). Type II was seen in 19% of teeth (5% to 33% in different studies).

An *ex vivo* study was performed in India. This study evaluated 220 first and 205 second molars. Almost 97% of first molars were three-rooted, and 48% had MB2 canal, which were mostly type IV. Also 93% of second molars were three rooted, 38% had MB2 canal, that mostly were type IV [22]. A study in China showed that 97% of the first molars were three rooted, and 52% had MB2. However, Vertucci’s classification was not evaluated in this study [23]. Another study performed evaluated 299 first and 210 second molars and reported that all first molars were three rooted with 52% presenting MB2 canal, that mostly were type IV. Second molars in 81% of case had three roots, 22% with MB2, mostly type IV [8]. The findings of the present study were similar to the other study conducted in China [23].

A recent study in Iranian population evaluated maxillary first molars in 250 CBCT images. All first molars in this study were three rooted, 86% had MB2 canal which were mostly type VI (about 35%) fallowed by types II, I, IV and V [17]. Type VI MB2 canal was not observed in the present study.

According to a recent review study evaluating the root anatomy and canal configuration of the second maxillary molar, most second maxillary molars had three separate roots [24]. MB2 canal was reported between 11.53 to 93.7% in different studies, with the predominance of type II in Brazilian and United state population, and types II and III in Chinese population [24]. In the present study, types I (57%) and II (39%) were observed in almost all second maxillary molars while no type III MB2 was reported.

The finding of the present study showed that MB2 canal is mostly present in first molar teeth, and more than two third of cases have a separate orifice. This result was in line with other studies [4, 6, 8, 22, 23]. The important issue to be considered is that, in the present study, most MB2 canals merge with MB1 canal along the root length (type II), while other studies reported type IV as the most prevalent Vertucci’s type. On the other hand, the results of present study showed that types II and I were the most frequent Vertucci types in second molars.

The relationship between the gender and tooth morphology is a controversial issue [25]. In the present study the difference in the frequency of MB2 in maxillary first molar was not significant between men and women. This is in agreement with some previous studies [26]. Considering second molars, the frequency was significantly higher in men. However, further studies with larger sample sizes are required to investigate if the gender has any effects on the frequency of MB2.

The present study found a correlation between the morphology of contralateral first or second molars, but the correlation was not high when comparing first and second molars. It indicates that when the clinician recognizes the morphology of a maxillary molar, the contralateral molar often has the same morphology. However, no prediction can be made about the ipsilateral molar. 

## Conclusion

In this selected Iranian population, the most common root morphology in maxillary first and second molars was three separate roots. MB2 was present in almost two third of first and less than half of second molars. In most cases, MB2 canal reaches the MB1 canal along the root length (Vertucci’s type II). The morphology and canal configuration of a maxillary molar can almost predict the morphology of contralateral molar, but not the ipsilateral molar. In the present study the root canal morphology, prevalence and type of MB2 canal was different from other ethnic groups.
